# MicroRNA-421-3p-abundant small extracellular vesicles derived from M2 bone marrow-derived macrophages attenuate apoptosis and promote motor function recovery via inhibition of mTOR in spinal cord injury

**DOI:** 10.1186/s12951-020-00630-5

**Published:** 2020-05-13

**Authors:** Jiaxing Wang, Yuluo Rong, Chengyue Ji, Chengtang Lv, Dongdong Jiang, Xuhui Ge, Fangyi Gong, Pengyu Tang, Weihua Cai, Wei Liu, Jin Fan

**Affiliations:** 1 Department of Orthopaedics, First Affiliated Hospital of Nanjing Medical University, Nanjing, 210029 Jiangsu China; 2 Department of Orthopaedics, Yancheng Third People’s Hospital, Yancheng, 224000 Jiangsu China

**Keywords:** Autophagy, Bone marrow-derived macrophage, mTOR, Spinal cord injury, Small extracellular vesicle

## Abstract

**Background:**

Spinal cord injury (SCI) has a very disabling central nervous system impact but currently lacks effective treatment. Bone marrow-derived macrophages (BMDMs) are recruited to the injured area after SCI and participate in the regulation of functional recovery with microglia. Previous studies have shown that M2 microglia-derived small extracellular vesicles (SEVs) have neuroprotective effects, but the effects of M2 BMDM-derived sEVs (M2 BMDM-sEVs) have not been reported in SCI treatment.

**Results:**

In this study, we investigated the role of M2 BMDM-sEVs in vivo and in vitro for SCI treatment and its mechanism. Our results indicated that M2 BMDM-sEVs promoted functional recovery after SCI and reduced neuronal apoptosis in mice. In addition, M2 BMDM-sEVs targeted mammalian target of rapamycin (mTOR) to enhance the autophagy level of neurons and reduce apoptosis. MicroRNA-421-3P (miR-421-3p) can bind to the 3′ untranslated region (3′UTR) of mTOR. MiR-421-3p mimics significantly reduced the activity of luciferase-mTOR 3′UTR constructs and increased autophagy. At the same time, tail vein injection of inhibitors of SEVs (Inh-sEVs), which were prepared by treatment with an miR-421-3p inhibitor, showed diminished protective autophagy of neuronal cells in vivo.

**Conclusions:**

In conclusion, M2 BMDM-sEVs inhibited the mTOR autophagy pathway by transmitting miR-421-3p, which reduced neuronal apoptosis and promoted functional recovery after SCI, suggesting that M2 BMDM-sEVs may be a potential therapy for SCI.

## Background

Traumatic spinal cord injury (SCI) has a very disabling central nervous system impact, which usually leads to irreversible sensory and motor impairment, and has a worldwide incidence of 15–40 cases per million people per year [1]. SCI involves two stages of pathological processes: primary injury and secondary injury [2]. Primary injury refers to the direct injury to the spinal cord; edema and hematoma of the spinal cord can further cause secondary injury. SCI is often accompanied by inflammation, apoptosis, autophagy, necrosis and oxidative stress, which can cause apoptosis of spinal neurons, especially neurons in the ventral horn, which may lead to loss of motor function [3, 4]. At present, surgery, drugs, hyperbaric oxygen and physical therapy have been widely used in clinical treatment of SCI and its sequelae, but none of them have achieved satisfactory results [5].

Small extracellular vesicles (sEVs) are extracellular vesicles with a diameter of 30–150 nm released by various tissues and cells [6]. By encapsulating DNA, messenger RNA (mRNA), microRNA (miRNA), proteins, lipids and other biologically active molecules, sEVs can mediate intercellular communication in a paracrine manner [7]. Recent research has focused on the content of sEVs to determine potential mechanisms in the treatment of various diseases [8–10]. Animal experiments have found that most of the sEVs are distributed in the liver and spleen after intravenous administration. Due to the presence of the blood–brain barrier, only a small number of sEVs actually enter the central nervous system [11]. However, spinal cord injury is accompanied by destruction of the barrier, and we speculated that the amount of sEVs entering the spinal cord would increase. In addition, several studies have shown that sEVs can enter the injured area after spinal cord injury and produce a significant therapeutic effect [12–14]. After injury, bone marrow-derived macrophages and activated microglia persist in the injured area for a long time, promote inflammatory progression and upregulate inflammatory factors [15]. At the same time, in the injury center, the permeability of damaged blood vessels increases, which aggravates the substantial infiltration of immune cells. Related research indicates that peripheral immune cells, especially M1 macrophages, enter the spinal cord after injury and produce deleterious effects. Depleting macrophages or enhancing the repair macrophage phenotype (M2 macrophages) can increase axon growth and improve motor function recovery [16, 17]. However, it is unclear whether sEVs derived from M2 macrophages can attenuate SCI.

Autophagy is an evolutionarily conserved and important cellular function in eukaryotes. It can remove misfolded proteins, protein aggregates and damaged organelles, and is a catabolic process that is necessary to preserve cell survival and homeostasis [18]. Studies have shown that autophagy is involved in the regulation of neuronal survival in neurological diseases and injuries [19, 20]. Inhibition of autophagy can increase neuronal apoptosis and cause neurodegeneration in mice [21], while enhanced autophagy promotes neurological recovery by inhibiting apoptosis [22]. Autophagy plays a key role in the regulation of neural function after SCI, and targeting autophagy may be a promising strategy for SCI treatment.

MiRNA is a class of endogenous non-coding RNA approximately 19–22 nucleotides long which can bind to complementary sites in the 3′-untranslated region (3′UTR) of its target mRNA and negatively regulate protein-coding gene expression, leading to protein degradation or translation inhibition [23]. In general, each miRNA can regulate hundreds of target genes and can participate in almost all cellular activities, including cell proliferation, differentiation, death and metabolism, among other functions [24, 25]. Recent studies have shown that miRNAs play a major role in mediating the effects of sEVs on recipient cells by affecting signaling pathways involved in the pathological response after SCI. Extracellular vesicle-encapsulated miRNAs play an important role in central nervous system functional recovery after SCI. In addition, miRNAs regulate autophagy by controlling autophagy-related proteins. MicroRNA-421-3p (miR-421-3p) is highly conserved among mammals and is involved in regulating a variety of disease processes such as tetralogy of Fallot [26], cancer [27] and bronchial dysplasia [28]. We found that mammalian target of rapamycin (mTOR) protein levels decreased after treating cells with M2 bone marrow-derived macrophage (BMDM)-sEVs. Bioinformatic analysis and miRNA sequencing results showed that M2 BMDM-sEVs contain miR-421-3p, which can target mTOR. However, it is unclear whether miR-421-3p regulates protective autophagy-mediated functional recovery after SCI by directly targeting mTOR.

In this study, we found for the first time to our knowledge that M2 BMDM-sEVs can protect neurons in SCI mice and promote motor function recovery. M2 BMDM-sEVs enhanced the autophagy level of neurons by inhibiting mTOR in vitro. In addition, we verified that reduced the activity of mTOR 3′UTR-luciferase reporter constructs. Intravenous injection of inhibitors of sEVs (Inh-sEVs), which were prepared by treatment with miR-421-3p inhibitor, showed lower protective effects than did injections of miR-421-3p mimic-containing sEVs (Mim-sEVs). Overall, our findings suggest that use of sEVs containing miR-421-3p may represent a new treatment for SCI.

## Results

### M2 BMDM and sEV extraction and identification

**Fig. 1:**
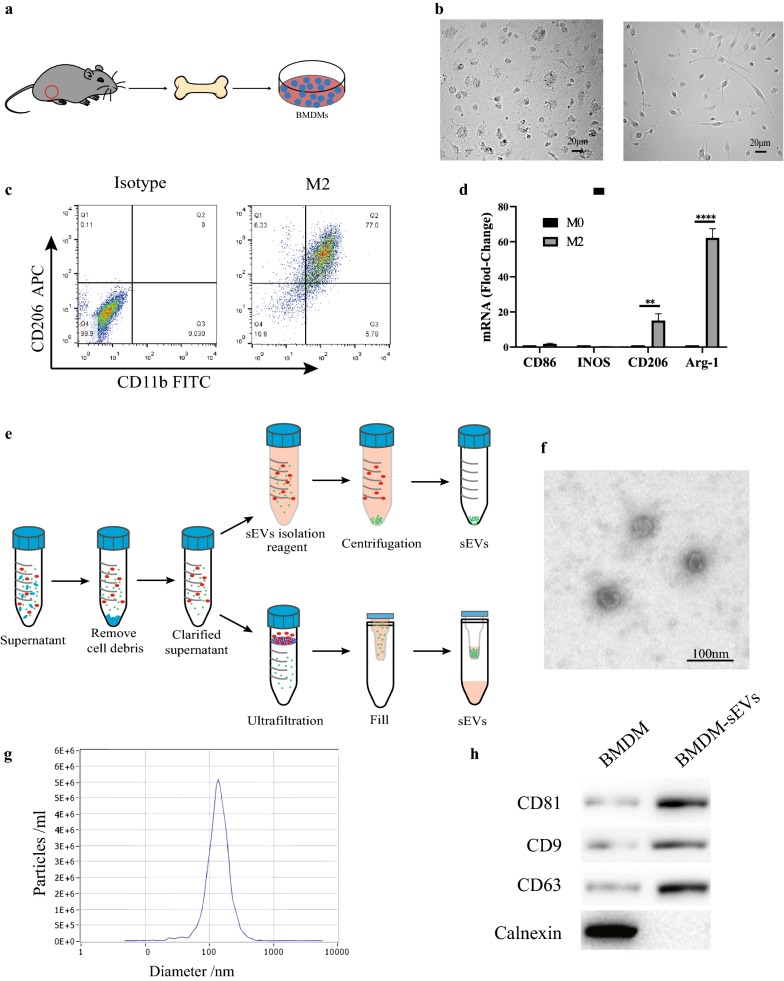
M2 BMDM and sEVs extraction and identification. **a** Flowchart of the isolation of BMDM. **b** M2 BMDM showed a typical spindle-type appearance. Scale bar: 20 µm. **c** Flow cytometry analysis of the macrophage markers on M2 BMDM, macrophage markers (CD11b) and M2 macrophage specificity markers (CD206) are highly expressed (**p < 0.01, ***p < 0.001). **d** QRT-PCR results of macrophage markers in induced M2 BMDM. **e** A schematic diagram of the extraction of BMDM-sEVs. **f** Morphology of BMDM-sEVs was observed by transmission electron microscopy (TEM). Scale bar: 100 nm. **g** Particle size distribution of BMDM-sEVs was measured by Nanoparticle Tracking Analysis (NTA). **h** Surface markers of BMDM-sEVs and BMDM was measured by western blotting (CD9: 25 kDa; CD81: 26 kDa; CD63: 26 kDa; Calnexin: 90 kDa)

First, we extracted BMDMs from the tibia and femur of mice and further induced M2 BMDMs (Fig. 1a). Next, we observed M0 BMDMs and M2 BMDMs under a microscope and the micrograph in Fig. 1b shows that M2 BMDMs had a typical spindle-type appearance. In addition, the results of flow cytometry experiments (Fig. 1c) demonstrated that the extracted cells highly expressed macrophage (CD11b) and M2 macrophage (CD206) surface markers, and the qRT-PCR results (Fig. 1d) show that compared with M0 BMDMs, the content of CD206 and Arg-1 (typical surface and intracellular markers of IL-4-induced M2 macrophages) was significantly higher in M2 BMDMs, indicating that we had successfully induced M2 BMDM. Next, we collected sufficient M2 BMDM supernatants to extract M2 BMDM-sEVs by ultracentrifugation and kit methods (Fig. 1e) and used TEM, Nanoparticle Tracking Analysis (NTA) and western blots to identify typical sEV features. TEM results showed that sEVs had a typical double-layer membrane structure with a diameter of about 100 nm, which is consistent with previous reports in the literature (Fig. 1f). Meanwhile, the NTA results also showed that the sEVs were about 30–150 nm in diameter (Fig. 1g). In addition, surface markers of sEVs (CD9, CD63 and CD81) were also expressed in western blot results (Fig. 1h). In summary, the results of the above experiments indicate that BMDMs can be induced into the M2 type and produce abundant sEVs.

### M2 BMDM-sEVs can be taken up by neurons and reduce Glu-induced cell apoptosis

**Fig. 2:**
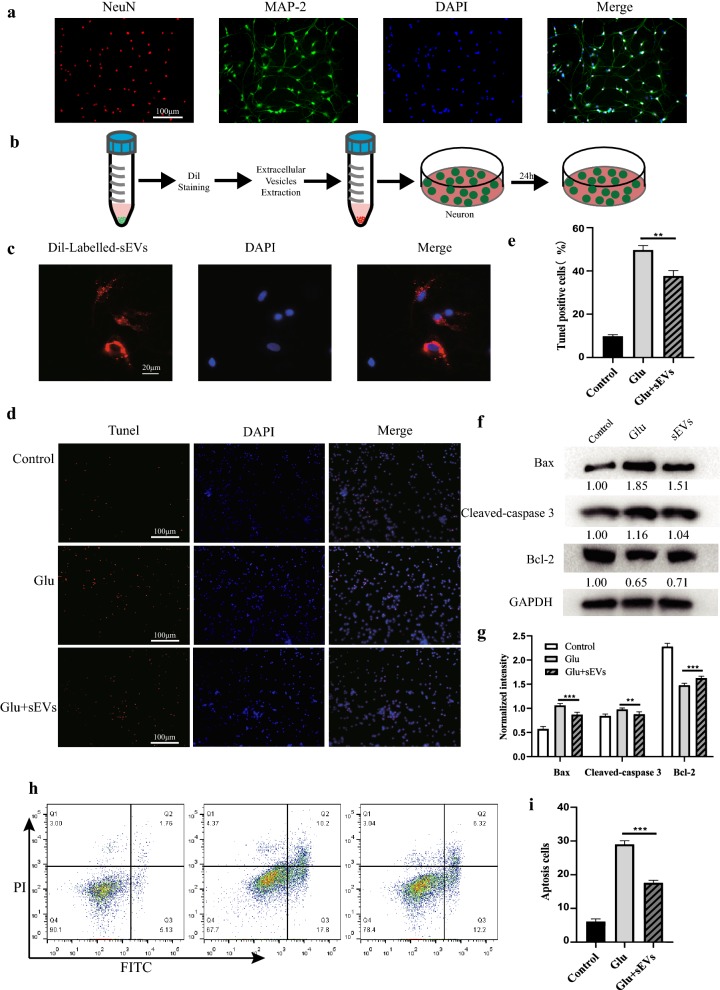
BMDM-sEVs can be taken up by neurons and reduce Glu-induced cell apoptosis. **a** Immunocytochemical identification of primary mice spinal cord neurons. Scale bar: 100 µm. **b** A schematic diagram of Dil-labeled BMDM-sEVs co-incubation with primary mice spinal cord neurons. **c** Representative Dil-labeled BMDM-sEVs in primary mice spinal cord neurons. Red fluorescence indicate Dil-labeled BMDM-sEVs and blue fluorescence indicate nucleus. Scale bar: 20 µm. **d** TUNEL staining (red) to detect the apoptotic cells in neurons. Cell nuclei were counterstained with DAPI (blue). Scale bar: 100 µm. **e** Quantitative estimation of the proportion of apoptotic cells in each group: control (untreated), glutamate (Glu) and BMDM-sEVs + Glu. BMDM-sEVs significantly reduced Glu-induced apoptotic cells, **p < 0.01. **f** Western blot analysis of apoptosis-related proteins in neurons. **g** Relative expression levels of apoptosis-related proteins normalized to GAPDH (**p < 0.01, ***p < 0.001). **h** Flow cytometry analysis of Annexin V/FITC/PI to detect the apoptotic cells in neurons. **i** Quantitative estimation of the proportion of apoptotic cells, ***p < 0.001

Neurons extracted from mice had typical neuron axon and dendritic morphology and could also be co-labeled with mature neuron markers MAP-2 and NeuN (Fig. 2a). Before co-culturing with neurons, we labeled M2 BMDM-sEVs with the red lipophilic fluorescent dye Dil (Fig. 2b). After incubating the neurons with Dil-labeled M2 BMDM-sEVs for 24 h (Fig. 2b), the Dil-labeled red fluorescent signal was observed using laser confocal microscopy. Dil-labeled M2 BMDM-sEVs were taken up by neurons and were mainly distributed in the cytoplasm (Fig. 2c). In addition, Western blot results showed that the content of sEVs marker protein in neurons increased after co-culture of sEVs (Additional file 1: Fig. S1a).

Our previous research [12] showed that Glu-induced neurotoxicity is one of the most important pathogenic mechanisms of neurological dysfunction after SCI, so it was used to produce an in vitro model of SCI-induced neuronal damage. First, we investigated whether M2 BMDM-sEVs exert a protective effect against Glu-induced neuronal apoptosis. As shown in Fig. 2d, e, the Glu-treated cells (100 μM, 30 min, 37 °C) had a significantly higher apoptotic rate (percentage of TUNEL-positive cells) than the control group, but pretreatment of cells with M2 BMDM-sEVs (100 μg/ml) for 24 h reduced the percentage of apoptotic cells, indicating that M2 BMDM-sEVs have a certain protective effect against Glu-caused neurotoxicity. As shown in Fig. 2f, g, the expression levels of the apoptosis-related proteins Bax and cleaved caspase-3 significantly increased after Glu treatment but decreased in neurons which were pretreated with M2 BMDM-sEVs. Meanwhile, the expression level of anti-apoptosis protein Bcl-2 was increased in the M2 BMDM-sEV pretreatment group compared with the Glu treatment group. In addition, we also used Annexin V-FITC/PI staining to detect the effect of M2 BMDM-sEVs on the apoptosis of Glu-treated neurons. As shown in Fig. 2h, i, the percentage of apoptotic cells significantly increased in cells treated with Glu (from 6.89 to 28.00%), while BMDM-sEVs partially reversed the effect of Glu (18.52%). Overall, the above results show that M2 BMDM-sEVs can be taken up by neurons and play a protective role in Glu-induced neuronal cell apoptosis.

### M2 BMDM-sEVs attenuate tissue damage after SCI and improve motor function recovery

**Fig. 3:**
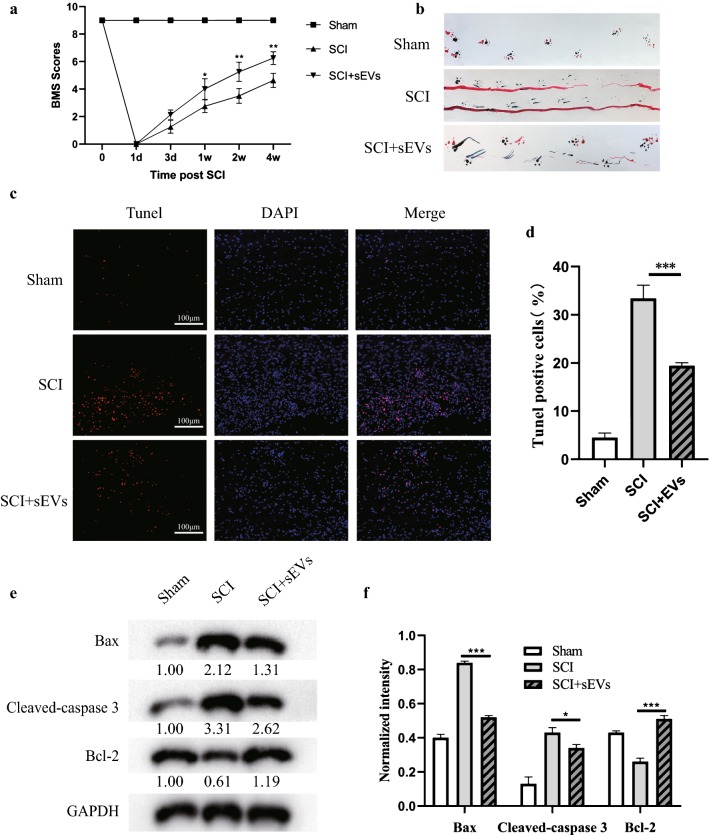
M2 BMDM-sEVs attenuate tissue damage after SCI and improve motor function recovery. **a** Basso Mouse Scale Scoring at different times after spinal cord injury. **b** Representative footprints of animal walking 28 days post SCI. Blue: front paw print; red: hind paw print. **c** TUNEL staining (red) to detect the apoptotic cells in spinal cord, nuclei were counterstained with DAPI (blue). Scale bar: 100 µm. **d** Quantification of TUNEL-positive cells in each group. **e** Western blot analysis of apoptosis-related proteins in spinal cord. **f** Relative expression levels of apoptosis-related proteins normalized to GAPDH (*p < 0.05, ***p < 0.001)

To further evaluate whether M2 BMDM-sEVs have neuroprotective effects in vivo, we established a mouse model of SCI at T10. SEVs injected into the tail vein can enter the injured area through the damaged blood spinal cord barrier and exert therapeutic effects (Additional file 1: Fig. S1b).As shown in Fig. 3a, BMS scoring showed that the SCI group and the sEV group completely lost their motor function on the first day, and began to recover at 3 days, but the BMS score of the sEV group was significantly higher than the SCI group at 1 week, and the gap between group scores continued to increase for 2–4 weeks. Next, we collected gait data from the sham group, the SCI group and the sEV group through footprint analysis (Fig. 3b). Compared with the sham group, mouse hind paw motor function was impaired after SCI, and the coordination of front and rear paw movements was significantly decreased; but the motor function of the sEV group recovered significantly, and their motor coordination was better.

We then used TUNEL staining to assess neuronal apoptosis in the SCI region. As shown in Fig. 3c, 3 days post-injury, sEVs significantly reduced the percentage of TUNEL-positive cells in the area of the SCI, which was consistent with the results of TUNEL staining in vitro (Fig. 3d). Finally, we used western blot to analyze protein expression changes after SCI (Fig. 3e, f). Consistent with the in vitro experiments, the expression levels of apoptosis-related markers Bax and cleaved caspase 3 were significantly increased in the SCI group, while the expression level of the anti-apoptotic protein Bcl-2, in the sEV group, was significantly higher than the SCI group. Taken together, these results demonstrate that M2 BMDM-sEVs can attenuate neuronal apoptosis and improve motor function recovery after SCI in mice.

### M2 BMDM-sEVs activate autophagy by inhibiting the mTOR signaling pathway

**Fig. 4:**
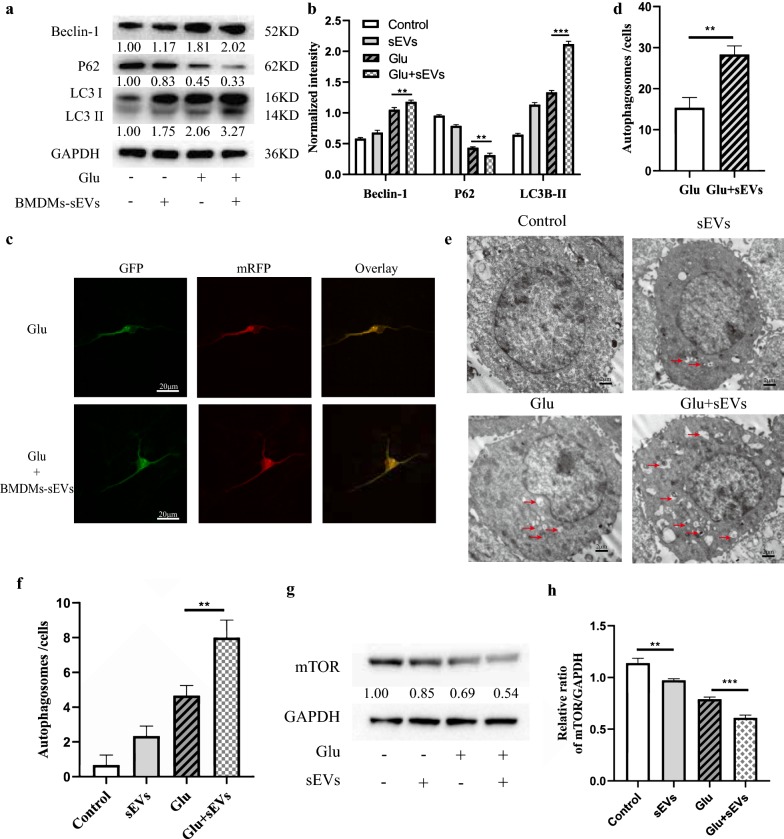
M2 BMDM-sEVs activate autophagy by inhibiting the mTOR signaling pathway. **a** Western blot analysis of autophagy-related protein in neurons. **b** Quantification of autophagy-related protein in each group. **p < 0.01; ***p < 0.001. **c** Autophagic flux of mRFP-GFP-LC3 transfected neurons was revealed by laser confocal microscopy. Autophagosomes are labeled by red and green fluorescence (yellow spots). Scale bar: 20 µm. **d** Quantification of autophagosomes numbers in each group. **p < 0.01. **e** Transmission electron micrograph (TEM) pictures of autophagosomes in neurons. Scale bar: 2 µm. **f** Quantification of autophagosomes numbers in each group. **p < 0.01. **g** Western blot analysis of mTOR protein expression in neurons. **h** Quantification of mTOR protein expression in each group. **p < 0.01; ***p < 0.001

Our previous study showed that autophagy plays an important role in maintaining neuronal survival after SCI. Therefore, we further investigated whether M2 BMDM-sEVs participated in the regulation of neuronal survival through autophagy. As shown in Fig. 4a, b, autophagy-related proteins LC3II and Beclin-1 gradually increased after sEV, Glu or sEVs + Glu treatment, while the P62 protein level gradually decreased. In order to observe the changes in autophagy flux more clearly and directly, the mRFP-GFP-LC3 lentivirus was used to transfect spinal neurons and its effects were observed using laser confocal microscopy. As shown in Fig. 4c, d, autophagosomes were labeled with red and green light (yellow fluorescence), while the BMDM-sEVs + Glu treatment group showed more yellow fluorescence than the Glu treatment group. In addition, TEM results (Fig. 4e, f) showed that using sEVs or Glu alone increased the number of autophagosomes, but the sEVs + Glu group showed more autophagosomes. Studies have shown that the inhibition of mTOR can induce autophagy [29], and mTOR-regulated autophagy can improve the symptoms of epilepsy and neuroinflammation in animal models of TBI, so we next investigated whether mTOR-regulated autophagy in the SCI mouse model also played a neuroprotective role. As shown in Fig. 4g, h, M2 BMDM-sEVs significantly reduced mTOR expression in neurons.

### M2 BMDM-sEVs reduce mTOR protein expression through transfer of miR-421-3p

**Fig. 5:**
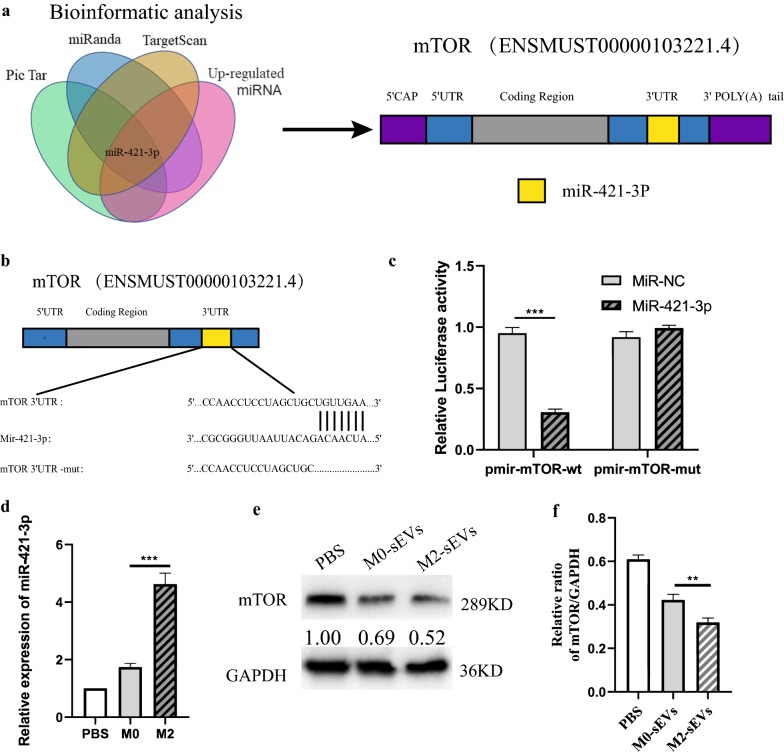
M2 BMDM-sEVs reduce mTOR protein expression through transfer miR-421-3p. **a** Bioinformatics analysis was used to predict the potential binding microRNA with mTOR. **b** Schematic representation of a predicted binding site of miR-421-3p in the 3′UTR of mTOR mRNA, and the mutated mTOR 3′UTR (mTOR 3′UTR-mut). **c** Dual-Luciferase Reporter System was used to detect luciferase activity. ***p < 0.001. **d** Neurons were treated with M0-sEVs or M2-sEVs for 24 h, then the relative expression of miR-421-3p was detected by qRT-PCR. **e** Western blot analysis of mTOR protein expression in neurons. **f** Quantification of mTOR protein expression in three groups. **p < 0.01

Recent studies have shown that sEVs can deliver encapsulated miRNAs to other cells to mediate intercellular communication and affect cell function. We therefore speculated that M2 BMDM-sEVs may reduce mTOR protein expression in neurons through miRNA transmission. First, three online bioinformatic analysis methods were used to predict miRNAs which targeted the 3′UTR of mTOR mRNA (Fig. 5a). Meanwhile, we sequenced the miRNA in M0 and M2 BMDM-sEVs and found that both M2 BMDM-sEVs and M0 BMDM-sEVs contained miR-421, and the content of miR-421 in M2 BMDM-sEVs was significantly increased. We then use the dual luciferase reporter system to determine the effect of miR-421 on mTOR 3′UTR activity in 293T cells. We first constructed luciferase reporter plasmids. The plasmid containing the wild type 3′UTR from mTOR was named pmir-mTOR and the plasmid containing a mutated mTOR 3′UTR region was named pmir-mTOR-mut (Fig. 5b). As shown in Fig. 5c, transfection of miR-421-3p mimics resulted in a significant decrease in luciferase activity in 293T cells transfected with pmir-mTOR, in contrast to cells transfected with the mutant 3′UTR. In addition, PBS, M0 BMDM-sEVs, and M2 BMDM-sEVs were used to treat the neurons for 24 h, and U6 was used as a control to determine the level of miR-421-3p by qRT-PCR. As shown in Fig. 5d, compared with neurons to which M0 BMDM-sEVs were added, the use of M2 BMDM-sEVs resulted in higher levels of miR-421-3p. Finally, we used western blot to detect the effects of M2 BMDM-sEVs and M0 BMDM-sEVs on the expression of mTOR protein in neurons. As shown in Fig. 5e, f, a significant decrease in mTOR protein expression was observed in neurons cultured with M2 BMDM-sEVs compared to M0 BMDM-sEVs. Overall, these results indicated that M2 BMDM-sEV-mediated downregulation of mTOR protein expression was dependent on miR-421-3p transmission.

### Upregulation of miR-421-3p reduces mTOR protein expression and enhances neuronal autophagy flux

**Fig. 6:**
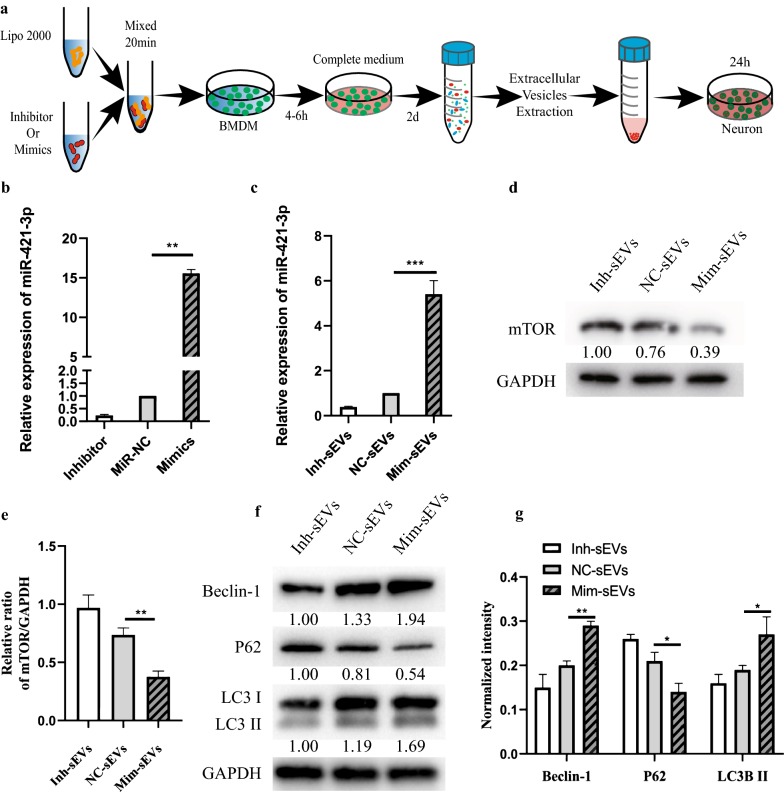
Upregulation of miR-421-3p reduces mTOR protein expression and enhances neuronal autophagy flux. **a** A schematic diagram of the extraction of NC-sEVs, Mim-sEVs and Inh-sEVs. **b** M2 BMDM was treated with miR-NC, mimics and inhibitor, then the relative expression of miR-421-3p was detected by qRT-PCR. **c** Neurons were treated with NC-sEVs, Mim-sEVs or Inh-sEVs for 24 h, then the relative expression of miR-421-3p was detected by qRT-PCR. **d** Western blot analysis of mTOR protein expression in treated neurons. **e** Quantification of mTOR protein expression in three groups. **p < 0.01. **f** Western blot analysis of autophagy-related protein in treated neurons. **g** Quantification of autophagy-related protein in each group. **p < 0.01; *p < 0.05

Next, we further studied the effects of miR-421-3p on mTOR and autophagy. As shown in Fig. 6a, we first transfected miR-421-3p mimics and inhibitor into M2 BMDMs. After 48 h, qRT-PCR results showed that the content of miR-421-3p in the Mimic group was significantly higher than in the miR-NC group, while the content of miR-421-3p in the Inhibitor group was lower (Fig. 6b). After transfection, we extracted NC-sEVs, Mim-sEVs and Inh-sEVs from miR-NC, Mimic and Inhibitor groups, respectively, which were used to treat Glu pre-treated neurons (Fig. 6a). As shown in Fig. 6c, Mim-sEVs significantly increased the content of miR-421-3p compared to Inh-sEVs. Meanwhile, western blot results showed that mTOR protein expression was lower after Mim-sEV treatment (Fig. 6d, e). Finally, we evaluated the effect of changing miR-421-3p content on protective autophagy. As shown in Fig. 6f, g, compared with the NC-sEV group, the expression of LC3II protein in the Mim-sEV group was significantly higher while expression in the Inh-sEV group was decreased. In conclusion, upregulation of miR-421-3p can enhance protective autophagy in neuronal cells by reducing mTOR protein expression.

### Tail-vein injection of Mim-sEVs increases autophagy flux and promotes motor function recovery

**Fig. 7:**
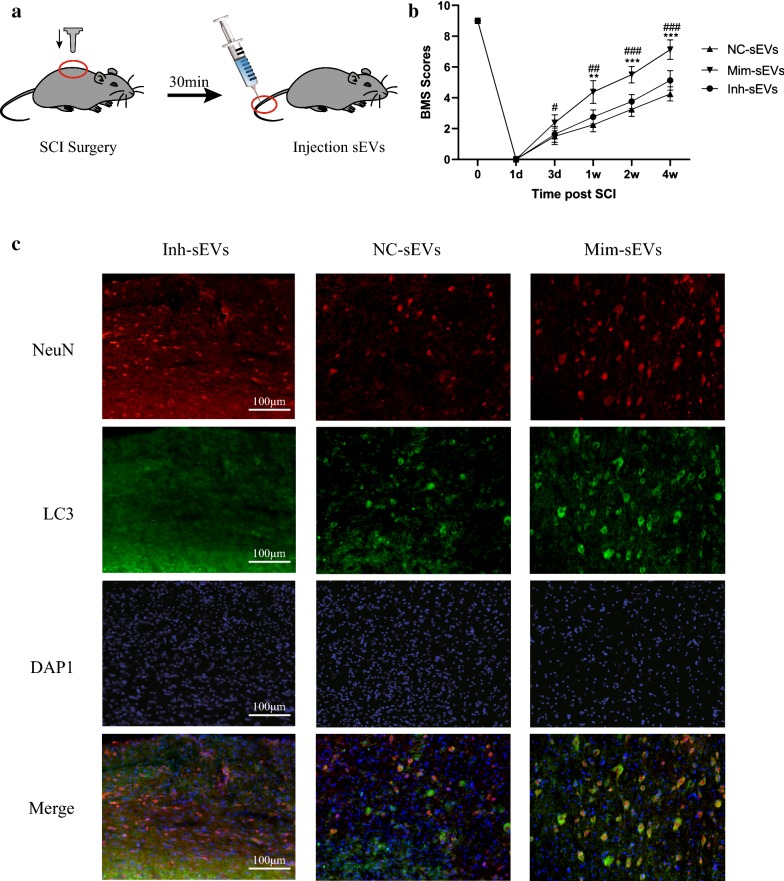
Tail-vein injection of Mim-sEVs increases autophagy flux and promotes motor function recovery. **a** Schematic diagram of the tail-vein injection of sEVs (200 μg). **b** Basso Mouse Scale Scoring for three groups at different times after spinal cord injury. Inh-sEVs: **p < 0.01; ***p < 0.001; NC-sEVs: ^#^p < 0.05; ^##^p < 0.01; ^###^p < 0.001. **c** Mice was treated with NC-sEVs, Mim-sEVs and Inh-sEVs 30 min post SCI, then the expression of LC3 (green) was detected by Immunocytochemical. Scale bar: 100 µm

To investigate whether miR-421-3p was involved in M2 BMDM-sEV-mediated autophagy regulation in vivo, NC-sEVs, Mim-sEVs and Inh-sEVs were used in the SCI mouse model. As shown in Fig. 7a, NC-sEVs, Mim-sEVs and Inh-sEVs were injected into the tail vein of SCI mice. Although BMS scoring showed motor function recovery in both the Mim-sEV and NC-sEVs groups, the BMS score of the Mim-sEV group was significantly higher than the other two groups. Furthermore, immunofluorescence staining showed that injection of Inh-sEVs had less effect on LC3 in the spinal cord of SCI mice (Fig. 7b, c). Taken together, these data indicate that protective autophagy induced by M2 BMDM-sEVs was dependent on the transmission of miR-421-3p.

## Discussion

SCI is still an unsolved medical problem [30], and despite decades of in-depth research, clinically effective treatments to restore function have not been developed. Both primary injury and subsequent secondary injury caused by SCI can cause severe nerve damage and irreversible loss of function. The main challenge that investigators face in the field of SCI is to develop effective therapies to prevent or reduce secondary injuries and promote neurological recovery after injury. The damaged spinal cord is difficult to regenerate, and chronic damage caused by the long-term presence of mononuclear macrophages and activated microglia in SCI sites often impairs functional recovery and results in progressive tissue degeneration [15]. SCI is often accompanied by the destruction of the blood spinal cord barrier, allowing blood-derived monocytes to migrate to the injury site and differentiate into macrophages 2–3 days after injury, which then participate in the regulation of injury with in situ activated microglia. Studies have shown that enhancing the macrophage phenotype or alternatively, transplantation of macrophages can increase axon growth, improve axonal transmission and promote functional recovery [18, 31, 32]. Although polarization or transplantation of M2 microglia/macrophages is a promising cell-based neuroprotective strategy, the optimal treatment parameters are difficult to assess. What is the effect of the blood spinal cord barrier on treatment? What dosage and treatment times should be employed for the use of drugs and cells? Do environmental factors during cell preparation and after transplantation lead to reduced or lost protective M2 function [33]?

SEVs are small vesicles secreted by cells and tissues which mediate intercellular signaling and regulate cell function by transporting biologically active molecules such as DNA, RNA, proteins and lipids [34, 35]. Previous studies have suggested that the therapeutic effect of polarized or transplanted cells may be due to their paracrine effects [36–38]. The use of sEVs in vivo avoids many side effects compared to drugs and cell transplants (for example, drug toxicity, low blood spinal cord barrier permeability, low cell survival rates, etc.). Song et al. [37] have shown that sEVs derived from M2 microglia have neuroprotective effects. However, BMDMs also participate in the regulation of central nervous system injury, so we alternatively hypothesized that M2 BMDM-sEVs would also have protective effects in SCI. To test our hypothesis, we conducted a series of experiments in vitro and in vivo. First, we extracted BMDMs from mice, then induced the M2 macrophage type. Flow cytometry and qRT-PCR results showed that we successfully extracted and induced M2 BMDMs. Next, we isolated sEVs from the supernatant of M2 BMDMs and identified the relevant characteristics of sEVs using western blot, TEM and NTA. Then, in vitro and in vivo SCI models were established and treated with BMDM-sEVs. It was found that M2 BMDM-sEVs reduced Glu excitotoxicity and promoted autophagy flux in secondary injuries. Previous studies have shown that autophagy helps maintain cell homeostasis and protects cells. This protective effect mainly includes the basic cell functions maintained by autophagy under physiological conditions and the inhibitory effect on apoptosis under pathological conditions [39]. Apoptosis is one of the main processes of secondary injury [40], and the number of neurons surviving after SCI determines the functional recovery. Our experimental results showed that in both in vivo and in vitro models of SCI, the application of M2 BMDM-sEVs reduced the apoptosis of neurons, and this decrease in apoptosis was mediated by autophagy. In order to confirm that BMDM-sEVs can reduce tissue damage by activating protective autophagy of cells, we further assessed the autophagy level of cells after M2 BMDM-sEV treatment. Western blot results showed that expression of LC3II and Beclin-1 (autophagy-related proteins) [41, 42] increased after M2 BMDM-sEV pre-treatment. At the same time, TEM and mRFP-GFP-LC3 lentivirus transfection results also showed that autophagosome numbers increased as did the autophagy level after M2 BMDM-sEV treatment.

MTOR is a serine/threonine protein kinase in the PI3K-related kinase family and plays a complex regulatory role in the physiology and pathology of the nervous system [43]. mTOR can form two distinct functional complexes, mTORC1 and mTORC2. mTORC1 stimulates protein synthesis and regulates autophagy, while mTORC2 is involved in the regulation of the actin dynamic skeleton [44]. Previous studies have shown that mTOR is a common autophagy regulator that is involved in the regulation of autophagy in multiple tissues [45, 46], and that inhibition of mTOR can play a certain neuroprotective role [44, 47, 48], so we speculated that M2 BMDM-sEVs may have activated cellular protective autophagy by targeting mTOR. Western blot results showed that M2 BMDM-sEVs significantly reduced mTOR protein expression. Next, we predicted the upstream miRNAs that targeted the mTOR 3′UTR and found that miR-421-3p was involved in the regulation of mTOR. Our sequencing results showed that miR-421-3p was highly expressed in M2 BMDM-sEVs, compared with M0 BMDM-sEVs. We then performed a series of experiments to verify the regulation of mTOR by miR-421-3p. qRT-PCR results showed that M2 BMDM-sEV-treated neurons had a higher miR-421-3p content than neurons treated with M0 BMDM-sEVs. Dual luciferase reporter tests and western blot results demonstrated that miR-421-3p could bind to the mTOR 3′UTR and inhibit mTOR protein expression. Finally, in the last set of experiments, we upregulated or knocked down miR-421-3p in M2 BMDMs and extracted three kinds of sEVs to treat in vivo and in vitro SCI models. The use of Mim-sEVs increased the content of miR-421-3p more than Inh-sEVs and induced a higher level of autophagy flux both in vivo and in vitro, resulting in improved motor function recovery.

## Conclusions

In summary, our study shows that sEVs from M2 BMDM-sEVs can enhance autophagy flux by directly inhibiting mTOR to promote functional recovery, which suggests the therapeutic use of extracellular vesicles to treat SCI.

## Methods

### Acquisition and induction of BMDMs

Cervical dislocation was used to euthanatize 4-week-old C57BL/6J mice, after which the femur and tibia were removed. A 1 ml syringe was used to flush out the bone marrow with cold phosphate-buffered saline (PBS). After passing through a 70-μm strainer, 5 ml of Red Blood Cell Lysis Buffer (Beyotime, China) was added to remove the red blood cells. After waiting 5 min, 15 ml PBS was added to the tube and mixed. The mixture was centrifuged for 5 min at 300*g* and the supernatant was discarded. The cells were then washed twice in PBS, resuspended in L-929-cell conditioned medium and cultured in Dulbecco’s modified Eagle’s medium (DMEM; Invitrogen, USA) containing 10% fetal bovine serum (FBS, Gibco, USA) and 1% penicillin/streptomycin (P/S, Invitrogen). The medium was changed every 3 days. On the 7th day, the mature BMDMs were cultured without L-929-conditioned medium for 24 h and defined as M0 BMDMs. Lipopolysaccharide (LPS, 100 ng/ml, PeproTech, USA) was used to stimulate the M0 BMDMs for 24 h and induce the formation of M1 BMDMs. Interleukin-4 (IL-4, 20 ng/ml, PeproTech) was used to stimulate the M0 BMDMs for 24 h and induce the formation of M2 BMDMs.

### Preparation of L-929 conditioned medium

Mouse L929 cells were diluted 1:10 and cultured in DMEM containing 10% FBS and 1% P/S. The conditioned medium was collected every 7 days, centrifuged at 1500 rpm for 5 min, filtered and stored at − 80 °C until use.

### Extraction and identification of M2 BMDM-sEVs

After co-culturing with IL-4 for 24 h, the M2 BMDMs were washed twice with PBS, then cultured in DMEM containing 10% exosomal-free FBS and 1% P/S. The supernatant was collected for extraction of sEVs after 2 days. We used two methods to extract sEVs, ultrafiltration and the ExoQuick™ kit (SBI, USA). The supernatant from M2 BMDMs was first centrifuged at 300*g* for 10 min and then centrifuged at 2000*g* for 10 min at 4 °C. The supernatant was filtered through a 0.22-µm filter (Steritop, Millipore, USA) to remove residual cell debris. In the kit method, the supernatant and extraction solution were mixed and allowed to stand for about 16 h at 4 °C, and then the mixture was centrifuged at 1500*g* for 30 min to obtain sEVs. In the ultrafiltration method, an Ultra-clear tube (Millipore) was used to centrifuge the supernatant (4000*g*, 40 min) until the volume in the upper compartment was approximately 200 µl. PBS was then added to the upper ultrafiltration compartment, the unit was ultrafiltered again to 200 µl, and the process was repeated once. M2 BMDM-sEVs collected by the different methods were stored at − 80 °C until use.

To identify the morphology of BMDM-sEVs, a transmission electron microscope (TEM, Tecnai 12, Philips, Best, The Netherlands) was used. Specific EV surface markers including CD81, CD9 and CD63 were analyzed by western blot. Nanosizer™ (Malvern Instruments, Malvern, UK) was also used to analyze the morphological characteristics of sEVs.

### Primary spinal neuron culture

After euthanatized C57BL/6J mice were immersed in 75% ethanol for 5 min, the entire spinal cord was dissected aseptically. The spinal cord was placed in cold DMEM/F12 medium (Thermo Fisher Scientific, USA) and the spinal cord membrane was removed. The spinal cord was cut into approximately 1-mm pieces, then transferred to a centrifuge tube with 0.25% trypsin (Thermo Fisher Scientific) and 0.05% deoxyribonuclease I (Sigma-Aldrich, St. Louis, MO, USA) to digest. After digestion in a 37 °C incubator for 20 min, horse serum (Sigma-Aldrich) was added to stop the digestion and the cells were centrifuged at 300*g* for 5 min and resuspended in DMEM/F-12 medium containing 10% horse serum, 0.5 mM glutamine (Thermo Fisher Scientific) and 1% P/S. After counting, neuronal cells were seeded into poly-d-lysine-coated 24-well plates or 6-well plates (Corning Inc, Corning, NY, USA) at a density of 5 × 10^4^ or 1 × 10^6^ cells/ml, respectively. After 4 h of incubation, the medium was replaced with neural basal medium supplemented with 2% B27 (Thermo Fisher Scientific), 0.5 mM glutamine and 1% P/S. One-half of the medium was replenished every 2 days. Immunostaining was performed after 7 days of incubation using antibodies against microtubule-associated protein 2 (MAP2; 1:500, rabbit IgG; Abcam, USA) and NeuN (1:800, mouse IgG; Abcam) to assess neuronal purity.

### BMDM-sEV uptake experiment

Following the manufacturer’s instructions, Dil solution (Molecular Probes, Eugene, OR, USA) was added to the sEV-containing solution (1:200) and incubated for 15 min at 4 °C. PBS was then added and the mixture was ultracentrifuged at 100,000*g* to remove excess dye, and this process was repeated three times. BMDM-sEVs that were fluorescently labeled were co-cultured with primary spinal neurons for 24 h, and the cultures were fixed with 4% paraformaldehyde for 15 min and washed three times with PBS. Finally, the uptake of BMDM-sEVs was observed by laser confocal microscopy.

### Flow cytometry

Cell suspensions were centrifuged at 300*g* for 5 min to collect BMDMs. The extracted BMDMs were resuspended in PBS and centrifuged, and this step was repeated twice to wash the cells. The cells were then incubated with FITC-conjugated anti-rat CD11b and APC-conjugated anti-rat CD206 antibodies (Invitrogen) for 30 min on ice. After washing twice with PBS, all samples were then analyzed by flow cytometry (FACSCalibur, BD Biosciences, USA). At least 5 × 10^5^ cells were analyzed from each sample.

Flow cytometry was also used to check the apoptotic rate. Glu- or sEV-pretreated neurons were harvested by centrifugation at 2000 rpm for 5 min. After washing twice with PBS, the harvested cells were resuspended in PI (5 μl, BD Biosciences) and FITC-labeled Annexin V (5 μl, BD Biosciences) for 5 min in the dark. After washing twice with PBS, the apoptosis rate was determined by flow cytometry.

### Terminal deoxynucleotidyl transferase dUTP nick end labeling staining

Our previous study indicated that glutamate (Glu, 100 µM) could be used to create an in vitro model of SCI-like cell death [12]. Primary spinal neurons were pretreated with BMDM-sEVs (100 μg/ml) or PBS for 24 h before Glu treatment. After fixing, rupturing and blocking, the cells were incubated with terminal deoxynucleotidyl transferase dUTP nick end labeling (TUNEL) solution (Roche, Basel, Switzerland) for 30 min in the dark according to the manufacturer’s instructions. After staining with DAPI (Beyotime) for 5 min, a fluorescence microscope (AXIO Vert. A1 and Imager A2; Carl Zeiss Microscopy GmbH, Jena, Germany) was used to observed TUNEL-positive cells. ImageJ (NIH, Bethesda, MD, USA) was used to calculate the proportion of TUNEL-positive (apoptotic) cells.

### Mouse model of SCI

We purchased 8-week-old female C57BL/6J mice (weight 18–22 g) from the Animal Center of Nanjing Medical University (Nanjing, Jiangsu, China). Mice are housed in groups (four per cage) in the laboratory animal center in a pathogen-free environment. The mice were maintained at room temperature (23 °C) under a light and dark cycle of 12:12 h with free access to water and food. This study was approved by the Ethics Committee of Nanjing Medical University. All procedures were performed according to the guidelines of the National Institutes of Health Animal Laboratory Animal Care and Use Guide.

Briefly, mice were anesthetized with isoflurane (2.0%, RWD Life Sciences, China) through the nasal cavity. After skin preparation and precise positioning, laminectomy was performed with the T10 spinous process at the center, and a 5 g rod (1 mm in diameter; RWD Life Sciences) was dropped from a height of 5 cm, causing spinal cord contusion. Animals were given preventive doses of antibiotics daily before and for 5 days after surgery, and animals received artificial bladder drainage daily after surgery until signs of normal bladder function were restored. Mice undergoing SCI surgery were randomly divided into two groups, the SCI group and the BMDM-sEV group (n = 8 per group). Sham group mice which only received laminectomy were used as the control group (n = 8). Mice in the SCI and BMDM-sEV groups were given PBS (200 μl) or BMDM-sEVs (200 μg total protein in 200 μl PBS), respectively, by tail vein injection 30 min post-SCI.

### Basso Mouse Scale Scoring

Motor function and hindlimb reflexes of mice after SCI were assessed by Basso Mouse Scale (BMS) scoring. Briefly, the mice were placed in an empty room and three trained experimenters assessed their motor function for 4 min after mice had adapted to the surrounding environment. The mice were scored for the severity of motor dysfunction on a scale of 0–9 (e.g., 9: near normal activity; 0: No ankle movement). Assessments were performed at 1 day, 3 days, 1 week, 2 weeks and 4 weeks after SCI.

### Footprint analysis

Assessment of mice gait and motor coordination was performed 4 weeks after SCI. The front and hind paws were painted with different dyes (red or blue). The mice were placed on a straight track with white paper at the bottom, and the animals were encouraged to move in a straight line. After collecting footprint pictures, the footprint pattern was digitized to assess the recovery of motor function.

### Western blot analysis

After extracting protein from cells and tissues, the protein concentration was measured using a BCA assay (Thermo Fisher Scientific) according to the manufacturer’s instructions. Proteins were separated by SDS-PAGE and transferred to polyvinylidene difluoride membranes. After blocking in 5% bovine serum albumin at room temperature for 2 h, the membranes were incubated with primary antibody overnight at 4 °C. After washing three times, the membranes were then incubated with horseradish peroxidase-conjugated anti-rabbit IgG or anti-mouse IgG antibody (1:2000, Thermo Fisher Scientific) for 2 h and visualized using enhanced chemiluminescence reagent (Thermo Fisher Scientific). ImageJ was used to assess protein expression levels. The primary antibodies used in this experiment were raised against CD9, CD81, CD63, Caspase-3 (all 1:1000, rabbit IgG; Cell Signal Technology, USA), Bcl-2, Bax, beclin-1, LC3B, P62 and GAPDH (all 1:1000, rabbit IgG; Abcam, USA).

### Immunofluorescence staining

Cells or tissue sections were fixed with 4% paraformaldehyde for 20 min, then permeabilized with 0.2% Triton X-100 for 20 min, blocked in 10% goat serum for 1 h and finally incubated with primary antibody overnight at 4 °C for dual immunofluorescence staining. The next day, the cells or tissue sections were incubated with Alexa Fluor 488 and Alexa Flour 594-conjugated goat secondary antibodies for 2 h at room temperature (1:200, Jackson ImmunoResearch, USA). After three washes with PBS, DAPI was used to counterstained the nuclei, and fluorescence images were acquired using a fluorescence microscope (AXIO Vert. A1 and Imager A2). Primary antibodies used were anti-MAP2 (1:500, rabbit IgG; Abcam, USA) and anti-NeuN (1:800, mouse IgG; Abcam, USA).

### TEM observation of autophagy

Pretreated neurons were collected, fixed with 2% glutaraldehyde solution for 2 h at 4 °C, then treated with 2% uranyl acetate solution for 2 h. After dehydration in 50%, 70%, 90% and 100% acetone, cells were embedded to make ultrathin sections for observation under an electron microscope (FEI Tecnai, Hillsboro, OR, USA).

### Double-labeled adenovirus mRFP-GFP-LC3 transfection

Primary neurons were seeded in a poly-d-lysine-coated confocal dish at a density of 1 × 10^4^ cells. After culture for 4 days, mRFP-GFP-LC3 lentivirus (Han Heng Biology, China) was used to transfect the cells. Then PBS, Glu, or BMDM-sEVs + Glu were added to different groups of cells. After fixing the cells with 4% paraformaldehyde, autophagy flux was observed by laser confocal microscopy (Zeiss LSM510, Oberkochen, Germany). Yellow spots represented autophagosomes and red spots represented autophagolysosomes.

### Prediction of potential binding miRNAs

In order to study the potential mechanism of sEVs reducing mTOR expression, three online bioinformatics methods [49–51] were used to predict potential miRNAs with binding sites for the mTOR mRNA 3′UTR. Among the predicted miRNAs, miR-421-3p had a higher content in M2 sEVs relative to M0 sEVs.

### MiR-421-3p mimic or inhibitor transfection

To overexpress miR-421-3p and inhibit miR-421-3p function, miR-421-3p mimic, inhibitor and corresponding negative controls (miR-NC or inhibitor-NC) were purchased from RiboBio Co, Ltd. (Guangzhou, China). M2 BMDMs were transfected by Lipofectamine^®^ 2000 (Invitrogen, Carlsbad, CA, USA) with a final concentration of 50 nM mimic or inhibitor following the manufacturer’s instructions. After transfection for 24–72 h, cells were harvested for miR-421-3p expression analysis and sEV extraction.

### Quantitative real-time PCR

Total RNA from cells or sEVs was extracted using TRIzol reagent (Invitrogen). The RNA was reverse transcribed into cDNA using a PrimeScript RT reagent kit (TaKaRa, Japan). The miScript SYBR Green PCR kit (QIAGEN, Germany) was used for quantitative real-time (qRT)-PCR. The relative expression of miRNAs was normalized to an internal reference (U6) and calculated using the 2−ΔΔCT method. Bulge-loop™ miRNA qRT-PCR Primer Sets (one RT primer and a pair of qPCR primers for each set) specific for miR-421-3p and U6 were designed by RiboBio (Guangzhou, China). Other primers used in this experiment included CD86 (forward: 5′-CTCAGTGATCGCCAACTTCA-3′, reverse: 5′-ATCTGCATGTTGTCGCCATA-3′), Inos (forward: 5′-AGGGACAAGCCTACCCCTC-3′, reverse: 5′-CTCATCTCCCGTCAGTTGGT-3′), CD206 (forward: 5′-GGGTTGCTATCACTCTCTATGC-3′, reverse: 5′-TTTCTTGTCTGTTGCCGTAGTT-3′), Arg-1 (forward: 5′-GGTTTTTGTTGTTGCGGTGTTC-3′, reverse: 5′CTGGGATACTGATGGTGGGATGT-3′) and GAPDH (forward: 5′-CCTTCATTGACCTCAACTACATG-3′, reverse: 5′-CTTCTCCATGGTGGTGAAGAC-3′).

### Luciferase reporter assay

We inserted the predicted target sites from wild-type mTOR 3′-UTR (pmirGLO-mTOR-WT) or mutated mTOR sequences (pmirGLO- mTOR-MUT) into the pmir-GLO-promoter vector (Promega, Madison, WI, USA). HEK293T cells were transfected with miR-NC or miR-421 mimic, then co-transfected with pmirGLO-mTOR-WT or pmirGLO-mTOR-MUT for 48 h. The dual luciferase reporter assay system (Promega) was used to measure luciferase activity in transfected cells.

### Statistical analysis

All experiments were repeated three times. We used IBM SPSS Statistics v17.0 and GraphPad 8.0.2 for statistical analysis. Student’s t-test and one-way ANOVA were used to calculate p-values. p < 0.05 was considered statistically significant (Additional file 1: Figure S1).

## Supplementary information

**Additional file 1: Figure S1.** (A) Western blot analysis of sEVs marker protein in sEVs-treated neurons or without sEVs -treated. (B) Representative Dil-labeled BMDM-sEVs in mice spinal cord. Red fluorescence indicate Dil-labeled BMDM-sEVs and blue fluorescence indicate nucleus. Scale bar: 20 µm.

## Data Availability

Not applicable.

## Abbreviations

SCI: Spinal cord injury
BMDM: Bone marrow-derived macrophages
SEVs: Small extracellular vesicles
mTOR: Mammalian target of rapamycin
MiR-421-3p: microRNA-421-3P
3′UTR: 3′ Untranslated region
PBS: Phosphate-buffered saline
DMEM: Dulbecco’s modified Eagle’s medium
FBS: Fetal bovine serum
P/S: Penicillin/streptomycin
LPS: Lipopolysaccharide
IL-4: Interleukin-4
TEM: Transmission electron microscope
TUNEL: Terminal deoxynucleotidyl transferase dUTP nick end labeling
BMS: Basso Mouse Scale
