# Supplementary figures and images for: MicroRNA-421-3p-abundant small extracellular vesicles derived from M2 bone marrow-derived macrophages attenuate apoptosis and promote motor function recovery via inhibition of mTOR in spinal cord injury
Source: J Nanobiotechnology. 2020 May 13;18:72. doi: 10.1186/s12951-020-00630-5 (PMC7222346; doi:10.1186/s12951-020-00630-5)

A

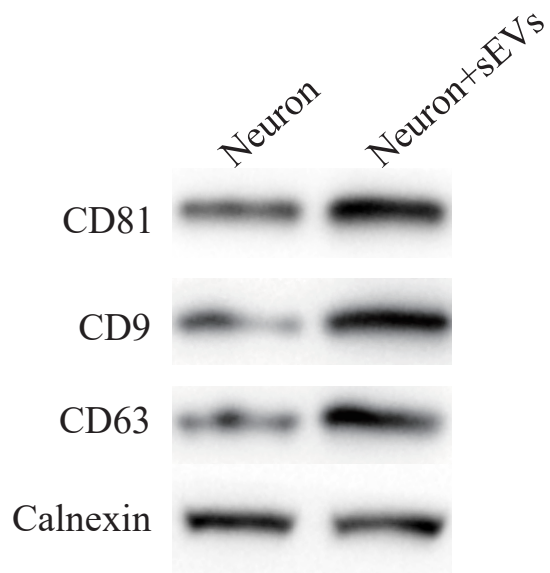

B

Dil-Labelled-sEVs

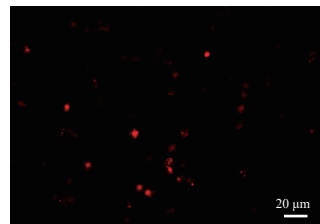

DAPI

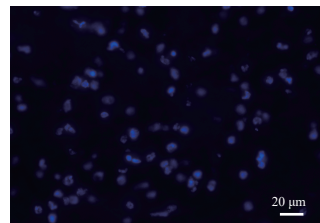

Merge

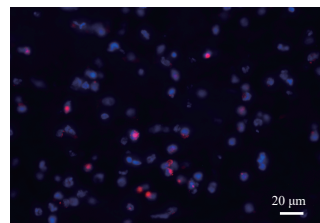

Supplement: Supplementary file 1 — Additional file 1: Figure S1. (A) Western blot analysis of sEVs marker protein in sEVs-treated neurons or without sEVs -treated. (B) Representative Dil-labeled BMDM-sEVs in mice spinal cord. Red fluorescence indicate Dil-labeled BMDM-sEVs and blue fluorescence indicate nucleus. Scale bar: 20 µm. [file 12951_2020_630_MOESM1_ESM.pdf]
